# Supplementary material for: HIVprotI: an integrated web based platform for prediction and design of HIV proteins inhibitors
Source: J Cheminform. 2018 Mar 9;10:12. doi: 10.1186/s13321-018-0266-y (PMC5845081; doi:10.1186/s13321-018-0266-y)
Supplement: Supplementary file 2 — Additional file 2. Source code of HIVProtI web server. [file 13321_2018_266_MOESM2_ESM.zip › HIVprotI_Source-code/hivprotI/weka-3-6-6/doc/weka/estimators/EstimatorUtils.html]

EstimatorUtils


---


|  |  |  |
| --- | --- | --- |
| **Overview** \| **Package** \| **Class** \| **Tree** \| **Deprecated** \| **Index** \| **Help** \| **Weka's home** | |  |
| **PREV CLASS**   **NEXT CLASS** | **FRAMES**    **NO FRAMES**     **All Classes** |  |
| SUMMARY: NESTED \| FIELD \| CONSTR \| METHOD | DETAIL: FIELD \| CONSTR \| METHOD |  |


---


## weka.estimators Class EstimatorUtils

```
java.lang.Object
  weka.estimators.EstimatorUtils
```

**All Implemented Interfaces:**: RevisionHandler

---

``` public class EstimatorUtils extends java.lang.Object implements RevisionHandler ```

Contains static utility functions for Estimators.

**Version:**
:   $Revision: 1.4 $

**Author:**
:   Gabi Schmidberger (gabi@cs.waikato.ac.nz)

---

**Constructor Summary**

`EstimatorUtils()`


| **Method Summary** | |
| --- | --- |
| `static java.lang.String` | `cutpointsToString(double[] cutPoints, boolean[] cutAndLeft)`             Returns a string representing the cutpoints |
| `static double` | `findMinDistance(Instances inst, int attrIndex)`             Find the minimum distance between values |
| `static Instances` | `getInstancesFromClass(Instances data, int classIndex, double classValue)`             Returns a dataset that contains of all instances of a certain class value. |
| `static java.util.Vector` | `getInstancesFromClass(Instances data, int attrIndex, int classIndex, double classValue, Instances workData)`             Returns a dataset that contains all instances of a certain class value. |
| `static Instances` | `getInstancesFromValue(Instances data, int index, double v)`             Returns a dataset that contains of all instances of a certain value for the given attribute. |
| `static int` | `getMinMax(Instances inst, int attrIndex, double[] minMax)`             Find the minimum and the maximum of the attribute and return it in the last parameter.. |
| `java.lang.String` | `getRevision()`             Returns the revision string. |
| `static void` | `writeCurve(java.lang.String f, Estimator est, double min, double max, int numPoints)`             Output of an n points of a density curve. |
| `static void` | `writeCurve(java.lang.String f, Estimator est, Estimator classEst, double classIndex, double min, double max, int numPoints)`             Output of an n points of a density curve. |

**Methods inherited from class java.lang.Object**

`equals, getClass, hashCode, notify, notifyAll, toString, wait, wait, wait`

**Constructor Detail**

### EstimatorUtils

```
public EstimatorUtils()
```


**Method Detail**

### findMinDistance

```
public static double findMinDistance(Instances inst,
                                     int attrIndex)
```

:   Find the minimum distance between values

    :   **Parameters:**: `inst` - sorted instances, sorted: `attrIndex` - index of the attribute, they are sorted after **Returns:**: the minimal distance

---


### getMinMax

```
public static int getMinMax(Instances inst,
                            int attrIndex,
                            double[] minMax)
                     throws java.lang.Exception
```

:   Find the minimum and the maximum of the attribute and return it in
    the last parameter..

    :   **Parameters:**: `inst` - instances used to build the estimator: `attrIndex` - index of the attribute: `minMax` - the array to return minimum and maximum in **Returns:**: number of not missing values **Throws:**: `java.lang.Exception` - if parameter minMax wasn't initialized properly

---


### getInstancesFromClass

```
public static java.util.Vector getInstancesFromClass(Instances data,
                                                     int attrIndex,
                                                     int classIndex,
                                                     double classValue,
                                                     Instances workData)
```

:   Returns a dataset that contains all instances of a certain class value.

    :   **Parameters:**: `data` - dataset to select the instances from: `attrIndex` - index of the relevant attribute: `classIndex` - index of the class attribute: `classValue` - the relevant class value **Returns:**: a dataset with only

---


### getInstancesFromClass

```
public static Instances getInstancesFromClass(Instances data,
                                              int classIndex,
                                              double classValue)
```

:   Returns a dataset that contains of all instances of a certain class value.

    :   **Parameters:**: `data` - dataset to select the instances from: `classIndex` - index of the class attribute: `classValue` - the class value **Returns:**: a dataset with only instances of one class value

---


### writeCurve

```
public static void writeCurve(java.lang.String f,
                              Estimator est,
                              double min,
                              double max,
                              int numPoints)
                       throws java.lang.Exception
```

:   Output of an n points of a density curve.
    Filename is parameter f + ".curv".

    :   **Parameters:**: `f` - string to build filename: `est` -: `min` -: `max` -: `numPoints` - **Throws:**: `java.lang.Exception` - if something goes wrong

---


### writeCurve

```
public static void writeCurve(java.lang.String f,
                              Estimator est,
                              Estimator classEst,
                              double classIndex,
                              double min,
                              double max,
                              int numPoints)
                       throws java.lang.Exception
```

:   Output of an n points of a density curve.
    Filename is parameter f + ".curv".

    :   **Parameters:**: `f` - string to build filename: `est` -: `classEst` -: `classIndex` -: `min` -: `max` -: `numPoints` - **Throws:**: `java.lang.Exception` - if something goes wrong

---


### getInstancesFromValue

```
public static Instances getInstancesFromValue(Instances data,
                                              int index,
                                              double v)
```

:   Returns a dataset that contains of all instances of a certain value
    for the given attribute.

    :   **Parameters:**: `data` - dataset to select the instances from: `index` - the index of the attribute: `v` - the value **Returns:**: a subdataset with only instances of one value for the attribute

---


### cutpointsToString

```
public static java.lang.String cutpointsToString(double[] cutPoints,
                                                 boolean[] cutAndLeft)
```

:   Returns a string representing the cutpoints

---


### getRevision

```
public java.lang.String getRevision()
```

:   Returns the revision string.

    :   **Specified by:**: `getRevision` in interface `RevisionHandler`
    :   **Returns:**: the revision


---


|  |  |  |
| --- | --- | --- |
| **Overview** \| **Package** \| **Class** \| **Tree** \| **Deprecated** \| **Index** \| **Help** \| **Weka's home** | |  |
| **PREV CLASS**   **NEXT CLASS** | **FRAMES**    **NO FRAMES**     **All Classes** |  |
| SUMMARY: NESTED \| FIELD \| CONSTR \| METHOD | DETAIL: FIELD \| CONSTR \| METHOD |  |


---
